# EpCAM an immunotherapeutic target for gastrointestinal malignancy: current experience and future challenges

**DOI:** 10.1038/sj.bjc.6603505

**Published:** 2007-02-27

**Authors:** M A Chaudry, K Sales, P Ruf, H Lindhofer, M C Winslet

**Affiliations:** 1 University Department of Surgery, Royal Free and University College London Medical School, Pond St, London NW3 2XA, UK; 2 Trion Research GmbH, Am Klopferspitz 19 D-82152, Martinsried, Germany; 3 Trion Pharma GmbH, Frankfurter Ring 193a D-80807, Munich, Germany

**Keywords:** EpCam, immunotherapy, trifunctional antibodies

## Abstract

Despite advances in surgery and adjuvant regimes, gastrointestinal malignancy remains a major cause of neoplastic mortality. Immunotherapy is an emerging and now successful treatment modality for numerous cancers that relies on the manipulation of the immune system and its effector functions to eradicate tumour cells. The discovery that the pan-epithelial homotypic cell adhesion molecule EpCAM is differentially expressed on gastrointestinal tumours has made this a viable target for immunotherapy. Clinical trials using naked anti EpCAM antibody, immunoconjugates, anti-idiotypic and dendritic cell vaccines have met variable success. The murine IgG2a Edrecolomab was shown to reduce mortality and morbidity at a level slightly lower than treatment with 5FU and Levamisole when administered to patients with advanced colorectal carcinoma in a large randomised controlled trial. Fully human and trifunctional antibodies that specifically recruit CD3-positive lymphocytes are now being tested clinically in the treatment of minimal residual disease and ascites. Although clinical trials are in their infancy, the future may bring forth an EpCAM mediated approach for the effective activation and harnessing of the immune system to destroy a pathological aberrance that has otherwise largely escaped its attention.

Gastrointestinal malignancy remains one of the largest causes of neoplastic mortality and morbidity. The use of immunotherapy to target tumours is well established. This combines antibodies that specifically target antigens selectively expressed on tumour cells and cytotoxic effector mechanisms. Successful clinical use includes the treatment of breast cancer by HER-2-specific trastuzumab (Goldenberg, 1999), follicular non-Hodgkin B-cell lymphoma by CD20 specific rituximab (Hainsworth, 2000).

Immunotherapeutic strategies for abdominal malignancy include targeting of the cell-adhesion molecule EpCAM. This article reviews the expression and function of EpCAM in normal and neoplastic tissue. The variety of strategies to target EpCAM are discussed, followed by a review of their use in clinical trials to target GI malignancy.

## EPCAM BIOLOGY

### Structure and function

EpCAM is a pan-epithelial differentiation antigen expressed on the basolateral surface of all carcinomas to varying degrees. As a homotypic cell adhesion molecule, it is intimately integrated within the Cadherin–Catenin and WNT pathways. It has recently been shown to modulate the expression of proto-oncogenes such as c-myc.

#### Tissue morphogenesis

**Figure 1:**
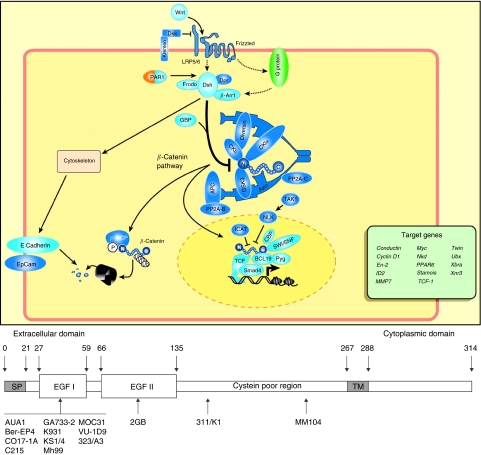
EpCAM domains and possible association of EpCAM with the Wnt pathway (modified from Huelsken and Behrens, 2002). EpCAM structure: extracellular, transmembrane and intracellular components are shown. The extracellular component contains three domains: the first is novel and is the site to which most of the antibodies developed are targeted (323/A3, 17-1A and others). The second is similar to EGF-binding proteins 1 and 6 and thyroglobulin. The third also has a novel structure that also has similarities with EGF. The intracellular portion of the antigen has a tyrosine phosphorylation site, the exact significance of which is uncertain.

EpCAM is essential for stable adhesion formation and tissue morphogenesis similar to adhesion molecules: carcino-embryonic antigen and ICAM-1. The precise mechanism by which cytoskeletal and intracellular elements mediate this function are being characterised. EpCAM inhibits intercellular adhesion mediated by cadherins (Litvinov *et al*, 1997) E-cadherin in turn interacts with *α*-, *β*- and *γ*-catenins forming the cadherin–catenin complex (Figure 1).

Catenins link cadherins with the actin cytoskeleton and form complexes with other proteins. Cadherins are crucial for the establishment and maintenance of epithelial cell polarity, morphogenesis of epithelial tissues and regulation of cell proliferation and apoptosis. Their association with *β*-catenin is particularly interesting as this is a component in the WNT pathway that regulates the expression of proto-oncogenes, such as c-myc: fundamentally associated with tumour development. Wnt glycoproteins are signalling molecules that regulate cell-to-cell interaction during embryogenesis. Wnt proteins bind to receptors of the Frizzled family. Through several cytoplasmic relay components, the signal is transduced to *β*-catenin, which is stabilised, accumulates in the cytoplasm, and enters the nucleus, where it binds a lymphoid enhancer factor/T-cell factor transcription factor. Together, *β*-catenin and lymphoid enhancer factor/T-cell factor activate expression of many target genes, such as *c-Myc*, VEGF, *cyclooxygenase-2*, all associated with neoplasia.

EpCAM directly impacts the cell cycle by upregulating c-myc and cyclin A/E. Human epithelial cells expressing EpCAM reduce growth factors dependency, and increase metabolism and colony formation. Inhibition of EpCAM expression with antisense mRNA reduces proliferation and metabolism in human carcinoma cells. The intracellular domain is essential for these effects (Munz *et al*, 2004).

EpCAM's adhesive properties promote calcium-independent homotypic cell sorting. Cells transfected to express EpCAM are sorted from cells of the same line that do not normally express EpCAM (Litvinov *et al*, 1994). It also inhibits invasive growth in cell colonies. Both activities are inhibited by anti-EpCAM antibodies (Litvinov *et al*, 1994).

### Pattern of tissue expression in the GI tract

#### Normal tissue

EpCAM is present in all normal epithelia excluding stratified squamous epithelia. Within the GI tract, colonic expression is the greatest and gastric the lowest. Glandular GI epithelium displays a marked expression gradient from crypts to the apex of villae.

#### Abnormal tissue

Carcinomas and actively proliferating tissues show that increased and differential expression of EpCAM.

Expression correlates with differentiation in gastric lesions. Immunochemical and mRNA studies show well-differentiated tumours are more expressive than those less differentiated. Normal background mucosa shows weak expression but, interestingly, areas of Barretts oesophagus or metaplasia are highly expressive (Litivinov, 1996; Wong, 2006).

90% of Colorectal carcinoma cells express EpCAM but in a differential form. Modifications include variable glycosylation analogous to tumour-specific antigens such as colonic tumour antigen MUC-1 (Denda-Nagai and Irimura, 2000). EpCAM exists in the cell membrane of colon carcinoma cells as a high-affinity noncovalent *cis*-dimer. Dimers on opposing membranes can associate via a head-to-head interaction to form tetramers with moderate affinity consistent with reversible intercellular associations. It is not known how exactly antibody binding correlates with variable glycosylation or oligomerisation in a functional or structural sense, and further investigation is required.

Tissue microarray studies allow comprehensive assessment of the tumour expression of EpCAM. A recent study compared 3900 tissues of tumour of stratified stages and grades of 134 different histological subtypes sourced from head and neck, lung, gastrointestinal, breast, urogenital and mesenchymal tumours. Seventy-five per cent tumour categories expressed EpCAM. At least weak EpCAM expression in >10% of tumours was observed in 87 of 131 different tumour categories. Colonic (81%), gastric, pancreatic (78%) and lung carcinomas revealed a high proportion of strongly positive tumours, suggesting EpCAM is an attractive target for pan-carcinoma immunotherapy (Went *et al*, 2004).

### Paradox of expression with advance in carcinomas

The functionally paradoxical upregulation of EpCAM with disease progression in colorectal, breast, prostate and upper GI carcinomas (Pocztek 1999; Gastl *et al*, 2000) remains unexplained. This is intriguing in metastatic carcinoma in which degradation of intercellular adhesions is a primary feature. Perhaps, EpCAM is upregulated in response to other intra-and extracellular processes that promote destruction of tissue adhesion and morphology, maintaining its constitutional stabilising function. Although this has not been investigated specifically, loss of EpCAM expression is clearly associated with increased local recurrence risk in patients with colorectal cancer (CRC) (Kapiteijn, 2002). In a study looking at resection specimens, loss of EpCAM was significantly associated with a diffusely infiltrative morphology but not with distant recurrence.

The relative loss of EpCAM expression in patients with gastric cancer is associated with a significant reduction in survival, indicating that loss of EpCAM expression identifies aggressive tumours especially in patients with stage I and II disease. Data from a Dutch study compared p53, CD44, E-cadherin, EpCAM and c-erB2/neu in tumours of 300 patients, investigating the extent of lymph node clearance. Patients without loss of EpCAM expression of tumour cells (19%) had a significantly better 10-year survival compared with patients with any loss: 42 *vs* 22%. The prognostic value was stronger in stages I and II, and independent of the TNM stage (Songun *et al*, 2005).

This presents yet another paradox suggesting that, once neoplastic transformation has taken place, a reduced EpCAM expression is an indicator of a more aggressive tumour phenotype with increased invasion, metastasis and mortality. This seemingly contradicts a recent study that suggested EpCAM silencing leads to reduced invasive potential of tumour cells. Breast cancer cell-lines were grown in Matrigel invasion/migration chambers. Cells in which EpCAM expression was silenced with SiRNAs showed a reduction of 35–80% in proliferation, 92% in cell-migration and 96% in cell-invasion without increase in cell death or apoptosis. There was, however, an increase in E-cadherin, *α*-catenin and *β*-catenin. This may be due to silencing of the inhibition that EpCAM exerts on E-cadherin. Alternatively, *EpCAM* gene silencing may lead to decreased cytoplasmic *β*-catenin through an increase in its association with the E-cadherin adhesion complex. Hence, reducing EpCAM may decrease *β*-catenin availability for the wnt pathway and activation of its target genes downstream (Osta *et al*, 2004).

We postulate that EpCAM expression increases up to a point after which destabilising factors predominate and its expression is no longer stimulated. Once this point is reached, which may be variable according to the particular tumour type, tumours are less stable and display greater invasive and metastatic potential. A comprehensive study of the temporal expression of EpCAM during tumour progression is currently lacking. This would be useful as a predictor of the efficacy of immunotherapy in individual patients according to their tumour stage.

Interestingly, a recent study revealed a 10-fold reduction in the expression of EpCAM in circulating tumour cells compared with primary tumours from whence they emerged and established metastases. (Rao, 2005). We postulate that the absence of homotypic adhesions in the vascular microenvironment may be the causal link. Indeed, EpCAM-targeted immunotherapy may be more effective in the context of established tumours or metastases as opposed to fluid-borne disease. This does not preclude ascites due to peritoneal metastases for which treatment with trifunctional antibodies is effective. However, the efficacy of destroying blood-borne circulating tumour cells in a hope of eradicating minimal residual disease may be limited.

## EPCAM TARGETED IMMUNOTHERAPY

Immunotherapy manipulates competent host immune system inducing tumour growth inhibition, regression or cytolysis. Approaches include the use of monoclonal antibodies and their derivatives, hybrid bispecific (trifunctional) antibodies, tumour cell vaccines, anti-idiotypic antibodies and dendritic cell vaccines. Pure immunomodulatory cytokines have been used to enhance the effect of monoclonal antibodies (MAbs).

### Mechanism of tumour inhibition

The mechanisms by which anti-EpCAM antibodies exert tumour inhibition *in vivo* remain controversial. Cytotoxic mechanisms include antibody dependent cell cytotoxicity (ADCC) mediated by natural killer cells and T lymphocytes, complement mediated cytolysis (CMC) and opsonisation promoting phagocytosis mediated by PMNs.

The question of whether anti-EpCAM antibodies directly inhibit tumour cell proliferation remains unanswered. It could be postulated that EpCAM antibodies directly interfere with the activation of the Wnt pathway causing downregulation of *c-myc*: this remains untested. The majority of anti-EpCAM antibodies produced are specific for epitopes within the first of two EGF-like domains in the extracellular segment of EpCAM (Figure 1), and none have been shown to mimic the dimerisation/tetramerisation that EpCAM undergoes on ligation or to interfere with downstream gene activation or cell proliferation *in vivo*. EpCAM antibodies do obliterate EpCAM mediated homotypic cell-sorting activity *in vitro*; this effect may be a competitive event preventing dimerisation alone. Although it is unlikely that a similar competitive effect takes place in established tumours, an investigation to see any effect on the establishment of metastases would be interesting.

We still await a comparison between the differences in the toxicity of antibodies according to the functional EpCAM domain targeted. It is possible that the majority of these antibodies work to opsonise cells alone – inducing the cytolytic mechanisms mentioned above – particularly as no physiological ligands for the extracellular domain of EpCAM other than EpCAM itself have been identified.

EpCAM has recently been found to form a complex with the tight junction protein Claudin-7 within its intra membranous segment. The physiological significance of this is not yet known, although an effect on apoptosis resistance in tumours is intriguing.

A flurry of interest followed the assertion that LAIR-1, a member of the inhibitory group of the immunoglobulin-like receptors, was a novel receptor for EpCAM. Speculation arose that neoplastic cells escape immunological surveillance and clearance by interacting with LAIR-1 via EpCAM gaining selective advantage for their growth, spread and dissemination. This seemingly attractive hypothesis was nullified when the original paper by Meyaard *et al*. was retracted because the observed binding of the LAIR-1 to EpCAM transfected cells was an artefact, attributed to the contamination of the LAIR-1 fusion protein preparation with an anti-human EpCAM monoclonal antibody.

#### Mechanism of ADCC

Both the direct antigen binding portion Fab and constant Fc regions of antibodies contribute to their activity. Fab portions bind to specific epitopes of target antigens whereas the Fc portion associates with immune effector cells to mediate ADCC, phagocytosis, endocytosis, release of inflammatory cytokines and antigen presentation. Both natural killer cells and monocytes express the FC receptor: Fc*γ*RIII also known as CD16 (Eccles, 2001).

Mice lacking activation Fc receptors (Fc*γ*RI-CD64 and *γ*RIII-CD16) show substantially reduced responses to antitumour MAbs. In contrast, mice deficient in the inhibitory receptor Fc*γ*RIIb-CD32 respond to the same antibodies with enhanced ADCC and tumour growth inhibition. Engineering the Fc portion to maximise Fc*γ*RIII binding may enhance the therapeutic benefit of immunotherapeutic agents. The significance in human context remains unknown (Clynes *et al*, 2000).

### Clinical trials

#### Monospecific murine antibody

*The murine Ig2a anti-human 17-1A monoclonal antibody:* (Table 1) Ederecolomab was the first immunotherapeutic agent licensed for use in large-scale human anti-tumour immunotherapy trials. Initial trials in patients with advanced CRC showed little improvement in morbidity or mortality. Augmentation with IFN and GM-CSF increased ADCC with associated tumour lymphocyte infiltration and complement deposition. Patients with greater ADCC survived longer.

In 1994, 189 patients with Dukes C CRC were randomly assigned to adjuvant therapy with Ederocolomab or resection alone. Survival at 3 years was 72% for the Ederocolomab cohort and 62% for surgery alone. Further follow-up at 7 years showed significantly reduced mortality (32%), disease recurrence (23%) and metastases leading to further phase II and III trials.

In 2002, Punt published results of a trial of 2761 patients randomised to MAb 17-1A monotherapy, 5-FU and Folinic-acid or 5-FU+Ederocolomab. No additional benefit was seen by adding immunotherapy to the standard chemotherapy regimen at 26 months. Immunotherapy alone was associated with significantly shorter disease-free survival; ederecolomab was removed from circulation.

The discrepancy between preclinical and clinical findings has led to much debate. What are the reasons for this discrepancy?

EpCAM expression density varies at different stages of tumour growth suggesting that patient antigen positivity should be assessed before clinical use. EpCAM density is a proven predictor of survival in breast cancer patients (Gastl *et al*, 2000).

As a murine antibody, Ederecolomab induces a neutralising humoural response in humans resulting in a short serum half-life. Foreign MAbs are rapidly cleared as immune complexes depositing in the liver, greatly reducing bioavailability. Reduced compatibility with human effector cells may also be significant (Pimm and Gribben, 1993).

EpCAM targeted immunotherapy to date has targeted advanced disease: its value weighed against classic adjuvant treatments. The effect of such immunotherapy on earlier, less established disease or cancer models is unknown.

What are the solutions?

#### Humanised antibody

A fully human IgG antibody, MT-201 combines binding affinity similar to Ederecolomab with considerably enhanced ADCC potency with human gastric carcinoma cell lines. Addition of human serum containing IgG or human peripheral blood monocytes halves MT201 ADCC but abolishes that of Ederecolomab: indicating the importance of human anti-mouse antibodies (HAMA) and compatibility of syngeneic effector cells (Naundorf *et al*, 2002).

MT201 reduces tumour growth in xenotransplanted HT-29 CRC cells in nude mice but only to a level similar to Ederecolomab. As any athymic model is deficient in effector cells, its utility in assessing effector responses comparing antibody type is limited. It is hoped human effector cells with greater type specificity of FC*γ* receptors will facilitate amplified tumour inhibition clinically. Trials are underway.

#### Anti-idiotypic antibodies

Active immunotherapies triggering specific T cells are being assessed. Strategies based on the administration of EpCAM antigen provided as DNA of the whole protein, or class I-HLA-binding peptides pulsed on DC or combined to adjuvants are also under investigation at preclinical or clinical level.

Anti-idiotypic antibodies attempt to stimulate a prolonged, active immune response. Immunisation with a primary antigen such as EpCAM generates anti-EpCAM antibodies termed Ab1. Anti-idiotypic antibodies are then generated against Ab1. These antibodies termed Ab2 mimic the tertiary structure of EpCAM. Ab2 antibodies are used as surrogate immunogens for the production of Ab3, which are active against the original antigen EpCAM. Ab2 are endocytosed by APCs and hence represented by major histocompatibility complex class II antigens to stimulate CD4+ T helper cells. This occurs in the presence of the co-stimulatory molecule CD80/86 on APCs interacting with CD28 on TH cells. These then proliferate and mature to lymphocytes secreting cytokines Il-2, IFN-*γ* and tumour necrosis factor (TNF)-*α*. Il-2 stimulate a humoral and cell-mediated immune response. The cell-mediated response causes CD8+ Cytotoxic T lymphocytes proliferation and subsequently mature under the influence of IFN-*γ* and TNF-*α* to mediate cytolysis either by Fas–Fas ligand interaction to cause apoptosis or by the exocytosis of vesicles containing perforins and other proteases.

Although the prolonged spectre of active immunity is attractive in theory, multiple trials assessing the efficacy of anti-idiotypic antibodies against EpCAM for advanced GI cancer have shown only marginal success (Table 1).

Among the first was an anti-idiotypic antibody targeted against the GA733-antigen associated with EpCAM (Co17-1A). Antigen-specific T-cell immunity was detected in all six patients immunized (Fagerberg *et al*, 1995).

Two trials were performed in 1994; the first developed rat anti-idiopathic antibody to EpCAM. Of nine CRC patients immunised, only 1 out of 3 developed active Ab3. A further trial assessed immunisation of 54 CRC patients using polyclonal goat and monoclonal rat Ab2 targeted against MAb CO17-1A (Ab1) prepared as alum precipitates. The majority produced Ab3 antibodies sharing epitopes with the corresponding Ab1. One out of three displayed delayed-type hypersensitivity and specific T cells that readily proliferated in the presence of antigen *in vitro* (Herlyn *et al*, 1994).

The immunogenicity of full-length EpCAM encoded by recombinant vaccinia virus (VV GA733-2) has been assessed in a murine model. This induces antibodies to previously unknown epitopes and promotes ADCC of CRC targets with murine macrophages. Immunized mice developed Ag-specific, proliferative and delayed-type hypersensitive lymphocytes. The proliferation of ras-transformed syngeneic tumour cells expressing human EpCAM was suppressed in vivo suggesting the potential of VV GA733-2 as a candidate vaccine for patients with CRC (Zaloudik *et al*, 2002).

## BISPECIFIC ANTIBODIES

### Structure and rationale for development

**Figure 2:**
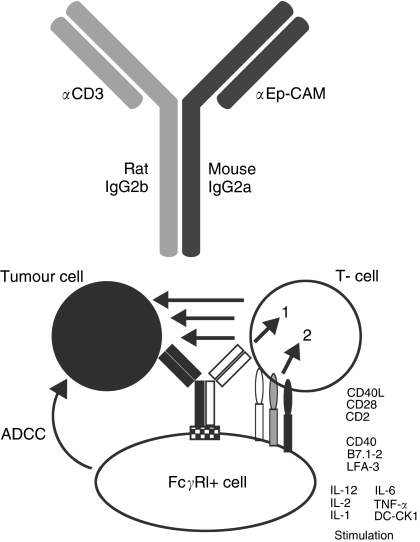
BiUII structure and functional advantage. The Fc portion of BiUII consisting of murine *γ*2a and rat *γ*2b heavy chains brings together two molecules that are highly potent activators of FcR similar to human *γ*1. BiUII has been shown to activate CD3+ lymphocytes, monocytes and macrophages expressing Fc *γ*RIII, NK cells expressing RI but not B cells expressing RII-CD32. These heterologous Fc portions were found to be more potent than the homologous Fc portions of either of the two parental antibodies used to construct BiUII.

Normal IgG molecules compose of Fc and FAb segments (Figure 2). The monospecific FAb segment binds to specific epitopes on antigens, whereas the Fc portion recruits cells expressing Fc receptors (e.g. Fc*γ*R) such as macrophages. These are described as being bifunctional and monospecific. In trifunctional antibodies, the two halves of the FAb segment have different specificity: they are bispecific and trifunctional.

Both Ederecolomab and MT201 are bifunctional antibodies: IgG1 and IgG2A respectively with active components being the anti-EpCAM FAb segments and Fc portions. Zeidler (1999) successfully constructed a bispecific/trifunctional, targeting both EpCAM and CD3 (BiUII or Removab). The rationale being that ADCC is complemented by the presence of CD3+ T-lymphocytes in addition to macrophage/monocytes, NK and dendritic cells, known to express Fc*γ*R binding to the Fc portion of the antibody. This antibody consists of a Murine IgG2a associated with an anti-EpCAM FAb segment and Rat IgG2b associated with anti-CD3 (Lindhofer *et al*, 1995).

### *In vitro* cytotoxicity

Initial *in vitro* experiments of ADCC with squamous cell carcinoma cell lines, effector cells and BiUII demonstrated increased production of IL-1*β*, IL-2, IL-6, IL-12 and DC-CK1. It is postulated that simultaneous stimulation of accessory cells and T lymphocytes leads to antigen presentation to T-lymphocytes inducing immunomodulation and cytotoxicity. BiUII induces production of IL-2 in the presence of EpCAM+ cells activating accessory and T cells without the usual requirement of exogenous IL-2. It is thought that an immunologically self-supporting tri-cell complex is formed, which is efficient for immune cell activation.

Interestingly, computerised sequential video microscopy shows that cytolysis occurs within 1–3 days, with no difference between patient and healthy donor PBMCs. The mode of cell death is characteristically necrotic and not apoptotic. Lymphocytes with pore-forming perforin proteins surround the tumour cells causing cytolysis (Riesenberg *et al*, 2001).

### Prolonged antitumour immunity *in vivo*

#### Another bispecific antibody

BiLu induces long-lasting antitumour immunity when administered intraperitoneally in a murine syngeneic model (Ruf and Lindhofer, 2001). It targets *murine* CD3 and human EpCAM. The Fc portion is identical to Removab.

The immunogenic function of BiLu, was investigated in an elegant study in which melanoma and A20 B-cell lymphoma cells were transfected with human EpCAM. Effective ADCC in the presence of BiLu and naïve spleen cells was greater for BiLu compared with its monospecific parental antibodies. Melanoma cells were injected into the peritoneal cavity of immunocompetent syngeneic mice, whereas B-cell lymphoma cells were introduced intravenously. Both groups received either intraperitoneal BiLu or parental antibodies.

All BiLu-treated animals survived; those treated with parental antibodies had significantly lower survival (100 *vs* 29%) and all tumour controls died by day 28. BiLu-treated surviving animals displayed an IgG2a humoral immune response but no IgG1 response. Nonsurvivors mounted only weak immune responses. Survivors rechallenged with reduced but potentially lethal doses of transfected cells established long-lasting immunity, actively rejecting tumour at day 144.

In a separate experiment irradiated, proliferation-incompetent transfected A20 cells and BiLu were used as an intraperitoneal vaccine. Tumour reactive antibodies were found in mice-administered BiLu but not in controls that had cells alone.

Both CD4+ and CD8+ T lymphocytes are required for the survival effects of the vaccination, generating humoral cytotoxic responses. Vaccination with antibody devoid of the Fc portion failed to induce immunity emphasising the indispensable nature of Fc*γ*RI+ cells.

Although BiLu is useful in murine models, its murine anti-CD3 specificity limits its role in human disease. Its counterpart Removab targets human CD3 and has shown promising results.

### Clinical trials

A phase I/II study for the treatment of ovarian cancer patients with symptomatic ascites has now been completed (23 patients), showing that Removab was safe and effectively reduced ascitic flow and tumour-cell content. A substantial phase II/III trial assessing efficacy in patients with all causes of malignant ascites including primary gastrointestinal tumours commenced in September 2004 (250 patients), and a Phase IIa study of platinum refractory ovarian cancer patients is also underway. Finally, a Phase I/II study of patients with peritoneal carcinomatosis owing to GI tumours but without symptomatic ascites is underway. Preliminary results are promising, indicating the utility of Removab in the treatment of minimal fluid borne disease and micrometastases.

## CONCLUSION AND FUTURE CHALLENGES

The discovery of antigens differentially expressed either on the surface of tumour cells or on part of their metabolic repertoire has created an opportunity for a new modality of cancer treatment. The concurrent development of monoclonal antibody technology has realised the potential of this opportunity.

Immunotherapy has the potential not only to provide magic bullets that trigger changes in the trajectory of cellular differentiation but also to harness with exquisite specificity the devastating storm of the immune system to eradicate tumours. Its potential for a long-lasting memory can also reduce recurrence.

The results of the largest clinical immunotherapy trials targeting EpCAM highlighted discrepancy between the high level of ADCC seen in vitro and disease-free survival and mortality. The classic problems of patient selection, time of therapy, delivery and specificity require further investigation.

Development of fully human MT201 antibody: currently being tested in phase I and II trials attempts to optimise delivery by alleviating HAMA production. Alternative approaches involving removal of the Fc portion retaining the Fab, F (ab)2 have been studied but only appear to be useful *in vitro*.

The functionally intricate role of EpCAM within the WNT pathway remains largely unexplored and therapeutically unexploited.

The temporal expression of EpCAM in the life cycle of human gastrointestinal tumours remains largely unstudied. Indeed, there may be a golden window of opportunity when EpCAM expression increases to a point after which metastatic tumour cells reduce expression. Use of EpCAM targeted immunotherapy earlier in disease progression may enhance tumour penetration and delivery to target cells in solid tumours.

Until we understand exactly how EpCAM antibodies on cancer cells, we cannot fully grasp what really happens *in vivo* and therefore we can only marginally improve EpCAM-based immunotherapy. Full structural information correlating the effect of antibodies on EpCAM structure, function and downstream pathways as well as gene regulation and proliferation require further research. Although it is widely held that ADCC is the major mechanism of tumour inhibition direct antibody effects *in vivo* cannot be precluded.

Through greater understanding of the Fc/FcR interplay of antibody and effector cells in cancer patients, it may be possible to engineer Fc portions of trifunctional antibodies with greater accessory cells specificity maximising ADCC.

Pre-clinical efficacy of bispecific/trifunctional antibody immunotherapy is proven. Utilisation of these exciting new antibodies to treat human solid carcinomas remains untested. The significance of human clearance of rodent antibody in the case of Removab remains unknown.

Clinical trials in this area are in their infancy. Preliminary results from intraperitoneal treatment of malignant ascites are promising. Indeed, the future seems exciting and may bring forth an EpCAM-mediated approach for the effective activation and harnessing of the immune system to destroy a pathological aberrance that has otherwise largely escaped attention.

## Figures and Tables

**Table 1:** Trials to assess efficacy of EpCAM targeted Immunotherapy for intra-abdominal carcinomas

**Author**	**Patients**	**Treatment**	**Results**	**Conclusions**
Weiner *et al* (1986)	27 Metastatic adeno carcinoma Colon or Pancreas	Passive MAb17-1A preceded by 4 days γIFN	No objective clinical markers Serum tumour markers reduced in 36% 11 developed Ab3 response	MAb 17-1A safe for clinical use Evidence of anti-idiotypic response
Herlyn *et al* (1994)	9 CRC	Active Anti-Idiotypic CO17-1A Aluminium hydroxide precipitated	3 patients developed Ab3 response to Ab2 determinants	Marginal success
Herlyn *et al* (1994)	54 CRC	Active Polyclonal goat and monoclonal Rat anti-idiotypic CO17-1A	Majority developed Ab3 response 30% developed delayed type hypersensitivity	Anti-idiotypic CO17-1A effective in stimulating log-term immunity in cohort
Fagerberg *et al* (1995)	6 CRC	Active Anti-Idiotypic CO17-1A	6 patients developed T-cell immunity 5 mounted Ab3 response	Small study Evidence of anti-idiotypic response
Ragnhammar *et al* (1995)	86 Adv CRC	Passive Murine MAb17-1A (76) or chimeric MAb17-1A (10)	All patients developed anti-idiotypic Abs Increased by GM-CSF; c-MAb less response and more allergic side effects than MAb	Patients with Ab2 response – median survival 9/12
Riethmuller *et al* (1998)	189 Dukes C	Passive observation or MAb17-1A Adjuvant	7 year evaluation, mortality decreased by 32% and recurrence by 23%	Therapeutic effect maintained after 7 years, mortality/recurrence reduced
Shetye *et al* (1998)	20 Adv CRC	Passive single infusion MAb17-1A+ GM-CSF	Increased tumoural and PMN, monocytes and T lymphocytes	Increased TILs representing ADCC and CTLs
Hjelm *et al* (1999)	20 Adv CRC	Passive MAb17-1A+IL-2+GM-CSF	1 patient partial remission 2 patients stable disease for 7 and 4 months	No augmentation of effect of MAb 17-1A
Punt *et al* (2002)	2761st III CRC	Passive Multicentre; (1) 17-1A MAb/5FU/LV or (2) 5 FU/LV or (3) 17-1A MAb	3-yr surv DFS (1) 74.7% 63.8% (2) 76.1% 65.5% (3) 70.1% 53.0%	Addition of Ederocolomab to standard therapy does not improve the disease outcome Panorex withdrawn
TRION Pharma, Fresenius 2003	23 symptomaticascites Ca Ovary	Passive trifunctional Multicentre open label intraperitoneal Removab	Well-tolerated 22 of 23 patients ascites free at day 37	Effective treatment of malignant ascites Phase III for all-cause malignant ascites underway
Heiss 2005	8 Peritoneal carcinomatosis	Passive Trifunctional 4-6 applications Intraperitoneal	7 of 8 patients no further paracentesis needed. Eradication of tumour cells in ascites	

ADCC=antibody-dependant cell cytotoxicity; CRC=colorectal cancer; CTL=cytotoxic T cells; DFS=disease-free survival; GM-CSF=granulocyte-macrophage-colony stimulating factor; IFN=interferon; MAB=monoclonal antibody; PMN=polymorphonuclear cells; TIL=tumour infiltrating lymphocytes.
